# Toward the novel AI tasks in infection biology

**DOI:** 10.1128/msphere.00591-23

**Published:** 2024-02-09

**Authors:** Artur Yakimovich

**Affiliations:** 1 Center for Advanced Systems Understanding (CASUS), Görlitz, Germany; 2 Helmholtz-Zentrum Dresden-Rossendorf e. V. (HZDR), Dresden, Germany; 3 Department of Renal Medicine, Division of Medicine, Bladder Infection and Immunity Group (BIIG), University College London, Royal Free Hospital Campus, London, United Kingdom; 4 Artificial Intelligence for Life Sciences CIC, Dorset, United Kingdom; 5 Institute of Computer Science, University of Wroclaw, Wroclaw, Poland; University of Michigan, Ann Arbor, Michigan, USA

**Keywords:** artificial intelligence, infection biology, deep learning, machine learning, bioimage analysis, host-pathogen interactions, natural language processing

## Abstract

Machine learning and artificial intelligence (AI) are becoming more common in infection biology laboratories around the world. Yet, as they gain traction in research, novel frontiers arise. Novel artificial intelligence algorithms are capable of addressing advanced tasks like image generation and question answering. However, similar algorithms can prove useful in addressing advanced questions in infection biology like prediction of host-pathogen interactions or inferring virus protein conformations. Addressing such tasks requires large annotated data sets, which are often scarce in biomedical research. In this review, I bring together several successful examples where such tasks were addressed. I underline the importance of formulating novel AI tasks in infection biology accompanied by freely available benchmark data sets to address these tasks. Furthermore, I discuss the current state of the field and potential future trends. I argue that one such trend involves AI tools becoming more versatile.

## INTRODUCTION

The past decade will, with all certainty, go down in history as the dawn of artificial intelligence (AI). Despite being largely mixed with dreams, hype, and fears, the family of computational techniques commonly referred to as AI has, without doubt, seen a significant development, as well as a surge in funding and adepts. As a field of computer science, AI aims to study and develop intelligent machines and software. With this, the ultimate goal of AI is to make highly general algorithms. Quite self-evidently it is making astonishing progress at it each year. Beyond the novelty, AI’s constituent parts like machine learning (ML) and deep learning (DL) are becoming more commonly used in research (1).

Yet, not all AI approaches are equal. While some ML techniques have been around for decades (2, 3), others like DL rely on the recent availability of advanced hardware. In return, DL and representation learning promise significant improvements in addressing biomedical data analysis tasks by learning relevant features (representations) in large quantities of data. As a rule of thumb, traditional ML algorithms fare well in a situation where structured data are available (e.g., a spreadsheet with quantifications or measurements), while representation learning and DL are useful for unstructured data (images, sequential continuous signal). Remarkably, in the case of biomedical image analysis, a number of community resources have been developed, including ZeroCostDL4Mic (4), DeepImageJ (5), or ImJoy (6). These resources lower the DL adoption hurdle for biologists. Furthermore, since the early adoption of DL in biomedical image analysis, significant improvements have been made in biomedical image segmentation (7, 8), the discovery of biomolecular interactions (reviewed in reference 9), and, perhaps most notably, the prediction of protein folding (10). The ability to address such advanced tasks is arguably what distinguishes the current generation of AI algorithms.

In an opinion issued almost 5 years ago (11), using two prominent contemporary studies (12, 13), I have described the surge of AI applications in infection biology as a tool for automating repetitive and laborious tasks. Undoubtedly, such a “sorting machine” is still the most widespread use case for AI/ML in infection biology. Yet, the forced closure of the economy of the world which occurred during the severe acute respiratory syndrome coronavirus 2 (SARS-CoV-2) pandemic has turned the world’s eyes to infection biology. For the data scientists of the world, detecting SARS-CoV-2 infection in patients’ chest X-ray images became a computer science exercise task (14–16) not dissimilar to the detection of handwritten digits (17). While numerous issues both from the side of biology and computer science exist with many models proposed in AI for coronavirus disease 2019 (COVID-19) (reviewed in reference 18), such transitions typically signify the AI maturity of the field. In other words, detecting infection no longer represents an exotic application for AI but is a widely accepted computer science problem to address.

Furthermore, in the past 5 years, something fundamental has changed about the state of AI in infection biology. The arrival of models like AlphaFold (10), large language model architectures (19, 20), and concepts like foundational models (FM) (21) is steering the field away from the “sorting machine” paradigm toward the “Swiss army knife” paradigm. This is exemplified by researchers and medical practitioners turning to general-purpose models like ChatGPT to address a great variety of tasks from writing a scientific abstract to writing a discharge summary (22, 23).

**Fig 1:**
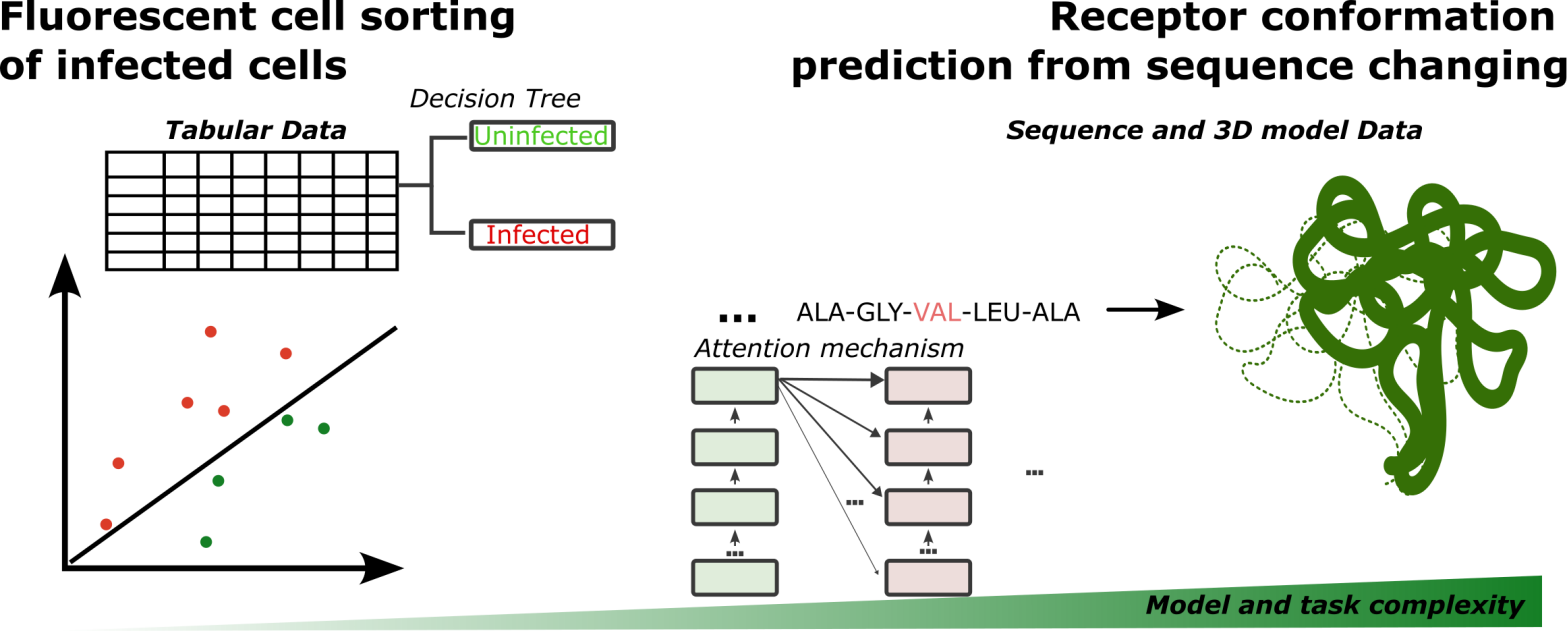
Illustration of artificial intelligence tasks in infection biology and their complexity. The left-hand side depicts the cell sorting task based on infection signal readout (e.g., fluorescence) addressable by simpler ML algorithms like decision trees. The right-hand side depicts a protein folding and conformation changes task requiring more complex algorithms like convolutional neural networks or transformers (here attention mechanism is illustrated for simplicity).

While analysis of structured databanks is arguably a more common ML application, modern AI algorithms strive to solve complex tasks in unstructured data (Fig. 1). This qualitative change hallmarks the next generation of AI/ML algorithms in research—a trend persisting in virology and microbiology. In this work, I will focus on examples of such tasks and data sets.

## AI FOR IMAGE-BASED INFECTION BIOLOGY DATA

Perhaps, one of the most obvious examples of unstructured data is images. They originate from drawings, photographs, micrographs, radiograph films, or more commonly today directly from digital cameras or scientific sensors. Digitally images are represented by matrices of pixels (or picture elements). In simple terms, digital images are arrays of brightness (single number) or color values (three or more values). Yet, a digital representation like this leads to the fact that similar objects may look different in scale and value, while different objects may look alike (e.g., a chest X-ray of a healthy person compared to a chest X-ray of a COVID-19 patient). Furthermore, if one is interested in understanding where a certain part of the image begins and ends (e.g., an organ) there may be no universal rules to perform such measurement. These challenges lead to image classification and segmentation tasks, which AI algorithms excel at. While biomedical images show no apparent structure, pixels are known to be spatially autocorrelated (24). This means that the value of an individual pixel depends on the pixel values of neighboring pixels. Hence, as long as the objects we are interested in are fairly large and properly sampled (25), algorithms like convolutional neural networks (CNNs) (1, 26) are extremely effective in analyzing them.

However, the high effectiveness of CNNs often comes at a price. In the case of shallower architectures (with less trainable parameters), models can be readily trained from scratch with hundreds to thousands of images (27). Yet, deeper architectures require vast amounts of training (or pre-training) data counting hundreds of thousands or millions of data points (28–30). In the case of infection biology, the SARS-CoV-2 pandemic provided an opportunity for collecting such vast data sets. For example, in the heat of the global emergency, multiple data sets were collated containing chest X-rays of alleged COVID-19 patients (31). This led to the creation of a great number of open data sets aimed at developing DL models for diagnostics of COVID-19 using chest X-ray images (reviewed in reference 32).

Unsurprisingly, hastily trained models, as well as imperfect, biased, and incoherent data sets (reviewed in reference 18), and other methodological caveats connected with developing medical devices did not lead to COVID-19 diagnostics using chest X-ray being a solved problem. However, arguably they did not have to. Instead, these efforts led to cementing infectious disease diagnostics as a formidable problem to solve for AI. Prior to this, most seasoned AI researchers and practitioners were unaware of tasks that could help address pandemics. Even if they were, the efforts of provisioning and annotating a data set that could address such tasks represented a major barrier. Therefore, collating and publishing such tasks and data sets (benchmarks) will undoubtedly lead to further advances in AI for infection biology. Furthermore, once the task is clear and widely known, over time, many existing issues with the data set may be addressed using more advanced algorithms. For example, improvement in detection models has been reported through the use of generative models like generative adversarial networks capable of balancing the data set through the generation of synthetic data points (33).

Beyond chest X-rays, the number of open data sets aiming to address open problems in infection biology is steadily increasing (34, 35). For example, Liou et al. released a data set containing clinical urine microscopy of urinary tract infection (UTI) patients aimed at UTI diagnostics (36). Spahn and colleagues recently published a large multi-task data set suitable for segmentation, object detection, denoising, artificial labeling, and super-resolution in the microscopy of bacteria (37). Kreis et al. proposed a latent space diffusion model to learn molecular conformations of SARS-CoV-2 spike protein directly from the cryo-electron microscopy imaging data set they collated (38). Furthermore, the last two works exemplify consolidation and transition to the “Swiss army knife” paradigm (Fig. 1). Additionally, having unified data sets to address a plethora of tasks makes them more attractive for computer scientists.

## AI FOR TEXT AND SEQUENCE-BASED INFECTION BIOLOGY DATA

Unstructured data can be sequential. Remarkably, since most biomolecules are oligomers or polymers due to their biochemical nature, it may be convenient to think of them as sequences (i.e., sequences of their constituent monomers). Such a way of representing biomolecules includes most nucleic acids (e.g., RNA and DNA), peptides, and proteins covering a lion’s share of biomolecules. Conveniently, due to the inherent necessity of writing down and distinguishing these molecules, biochemists and molecular biologists have already come up with a way of codifying sequences of genes and proteins by their respective bases and amino acids. Furthermore, a similar code also exists for small molecules through the Simplified Molecular-Input Line-Entry System (SMILES) (39).

Representing molecular data sets as sequences makes them compatible with a number of powerful AI algorithms including recurrent neural networks like long short-term memory networks (40, 41) and transformers (19). These algorithms open doors to formulating learning tasks aimed at understanding patterns in molecular interactions. In the context of infection biology, interactions between molecules of the host and the molecules of the pathogens define the majority of the processes occurring during the infection and are decisive for its outcomes (42). Immersed in the environment of the host many pathogens strongly rely on such interactions. Therefore, understanding host-pathogen interactions (HPI) and being able to predict them often allow researchers to find cues to combating the disease.

One prominent kind of HPI is protein-protein interactions (PPI). PPI prediction has meanwhile established itself as an important task for ML in infection biology. For example, Karabulut and colleagues constructed a highly curated PPI data set and employed it to develop adenovirus PPI predictive models with a handful number of classifier algorithms (43). Ray et al. employed curated drug-protein and protein-protein interaction data combined with SARS-CoV-2 protein interaction data. The authors proposed a deep variational graph autoencoder to learn the structure of the resulting encompassing molecular interaction network and predict missing links. This allowed them to develop models detecting novel targets for host-directed therapy against COVID-19 (44).

Perhaps surprisingly, yet another example of unstructured data is natural language; more specifically, written text. Despite the simplicity of the digitalization of written language as well as the vast amount of text data online, language is far from being structured. It is full of ambiguities arising from the use of professional jargon and homonyms (45). Yet, throughout the history of the field of natural language processing (NLP), vast data sets (corpora) have been accumulated. Among them is the open-source data set of biomedical research papers collected by PubMed (46). This data set is widely used for a great variety of NLP tasks including text summarization (47) as well as general-purpose transformer pre-training (48). Furthermore, the vast amounts of social media data have proven resourceful in analyzing real-world data of post-acute COVID-19 (49). Bringing natural language and sequences together, Köksal and colleagues developed an AI-based search engine for related proteins in literature opening another avenue for PPI prediction (50). Beyond using conventional NLP techniques, purpose-built architectures for genetic sequences can explore relevant biology or be biology-informed. One such example includes an architecture employing equivariance to account for reverse compliments in genetic sequences (51).

## MULTIMODAL AI FOR INFECTION BIOLOGY DATA

Tying it all together, advanced AI tasks do not have to come from one particular data source or type. Once any input data format is converted into its vector representation, such representation can be combined, for example, in the *n*-dimensional latent space (reviewed in reference 52). Along these lines of thought, a recent work by Zhang et al. proposed a technique integrating image-based data and omics (53). Furthermore, Cantini and co-authors recently compared several methods for joint dimensionality reduction applicable to multi-omic data. With this, the authors aimed at eliciting the most optimal approach for the integration of multiple sources of knowledge (54).

With the wealth of open data sets available, this realization is rapidly spreading in the biomedical domain in general, as well as infection biology in particular. For example, Kuchroo and colleagues have recently proposed the potential of heat diffusion for affinity-based trajectory embedding, a method that can learn and visualize abstract cellular features and groupings in data types including flow cytometry, single-cell RNA sequencing (scRNA-seq), single-cell sequencing assay for transposase-accessible chromatin (scATAC-seq), and clinical variables (55). Another exemplary effort from Chen et al. consolidates medical multimodal data including physiology, omics, laboratory tests, imaging pathology, symptoms, and other clinical data (56).

However, it is worth mentioning that the intuitive benefits of multimodal efforts may sometimes turn out to be much less than the sum of the respective components. After all, the multimodal moments are as weak as the weakest representation. Furthermore, the multimodal models are as noisy as the noisiest data set.

## CONCLUSIONS

It would hardly be an overstatement to say that the infection biology community often shies away from digital trends. Despite the obvious progress in the field of AI, finding AI tools in an average infection biology laboratory might have been hard just a few years ago. Since the appearance of tools like ChatGPT, this changed quite dramatically. AI algorithms are now more sophisticated than 5 years ago and are capable of solving much more advanced tasks. However, the path to this state involved collecting large data sets and formulating complex tasks like question answering and text summarization as AI problems.

In this work, I discussed several examples of advanced AI tasks in infection biology that use unstructured data like images, genetic sequences, protein sequences, and scientific texts. In many cases, these tasks come with large curated data sets facilitating the development of novel algorithms in the infection biology community. At this point, it is tempting to imagine that these trends will lead to two major trends. One will involve the development of more advanced tools for infection biology akin to predictions of SARS-CoV-2 spike protein confirmations (38). The other trend will make the infection biology data sets and benchmark known and available to the data science community. This in turn will make more general-purpose tools like FM capable of solving tasks the infection biology community is interested in, thereby accelerating the research.

Regretfully it took a global pandemic to get us to this point. However, as the global COVID-19 cases dashboards get forgotten, we must continue the efforts of curating the data sets and challenging the status quo. Making sure that large open data sets are available is key to the next step in the evolution of novel AI tasks in infection biology.
